# Mental health burden of conflict: rates and correlates of depressive and anxiety symptoms among displaced Palestinian children and adolescents in Qatar

**DOI:** 10.1192/bjo.2025.10912

**Published:** 2025-12-05

**Authors:** Mohamed Adil Shah Khoodoruth, Yahia Albobali, Olfa Selmi, Sami Ouanes, Marwan Abdelkarim Ali Abdelkarim, Areeg Hassan Mohamed Elhassan, Menatalla Abdelkader, Taieb Turki, Ahmed Abdelhakim Ahmed Elzok, Abdul Waheed Khan, Majid Alabdulla, Yasser Saeed Khan

**Affiliations:** Hamad Medical Corporation, Doha, Qatar; College of Medicine, Qatar University, Doha, Qatar; Schulich School of Medicine & Dentistry, Western University, London, Canada; MindWell, Kuwait City, Kuwait

**Keywords:** Anxiety disorders, depressive disorders, war, displacement, children

## Abstract

**Background:**

Children displaced by armed conflict are at high risk of experiencing psychological distress. The ongoing war in Gaza has resulted in widespread trauma among Palestinian youth, yet limited data exist on their mental health following displacement. This study assessed the prevalence and correlates of anxiety and depressive symptoms among war-displaced Palestinian refugee children and adolescents resettled in Qatar.

**Aims:**

To estimate the prevalence of clinically significant anxiety and depressive symptoms and to identify psychosocial and trauma-related factors associated with symptom severity in this population.

**Method:**

A cross-sectional study was conducted among 350 Palestinian children (aged 8–17 years) residing in a residential compound in Qatar. Symptoms of anxiety and depression were measured using the Screen for Child Anxiety Related Emotional Disorders-Child Version and the Short Mood and Feelings Questionnaire-Child Version, respectively. A Resilience and Demographic Questionnaire was devised to assess trauma exposure and psychosocial variables. Multiple linear regression identified factors associated with symptom severity.

**Results:**

Clinically significant anxiety and depressive symptoms were found in 70.9 and 46.0% of participants, respectively. Separation anxiety was the most common subtype. Female gender, witnessing death, physical injury and disrupted caregiving were significantly associated with worse outcomes.

**Conclusions:**

This study highlights the urgent need for trauma-informed, culturally sensitive mental health services for displaced Palestinian children and young people. While clinical interventions are vital, a sustainable resolution to the conflict is essential to mitigate further psychological harm.

## Graphical abstract

**Figure:**
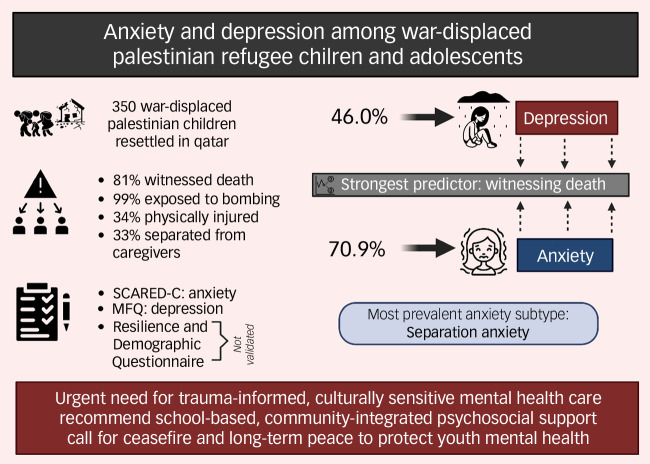

Children are disproportionately affected by global conflict and displacement, comprising one-third of the world’s population yet accounting for half of all refugees.^1^ Alarmingly, one in six children lived in conflict zones in 2020.^2^ Armed conflicts cause severe mental health consequences in children, including trauma, post-traumatic stress disorder (PTSD), anxiety and depression, further exacerbated by displacement, loss of caregivers and exposure to violence.^3^ In conflict zones, mental health impacts include sharply increased rates of PTSD, anxiety and depression, with prevalence two to four times more than the global average, with women and children being disproportionately affected.^4^ In addition to the direct effect of war, indirect effects, which are demonstrated in daily life stressors, such as poverty, family violence, famine and severe food insecurity^5^ often have a detrimental impact on children’s mental health.^6^

The protracted Israeli-Palestinian conflict has caused significant Palestinian displacement and created enduring socioeconomic challenges.^7^ Recent escalations in violence, including airstrikes and ground operations, have exacerbated psychological distress, particularly among children. Violence, forced displacement because of military action and bereavement have substantially increased mental health risks for this vulnerable population.^8^ Existing research has extensively documented the mental health burden among Palestinian children.^9,10^ Epidemiological data reveal disproportionately high rates of PTSD and depression in this population compared with both global and regional benchmarks. As highlighted by Shukri and colleagues, while global prevalence estimates for depression and PTSD remain relatively low, Palestinian children demonstrated higher rates, underscoring the disproportionate psychological burden faced by youth in conflict-affected settings.^11^

This study aims to evaluate the rates of anxiety and depression in war-displaced Palestinian children and adolescents, who are affected by the current war and who reside in Qatar. As part of a humanitarian response, Qatar launched a programme in December 2023 to provide medical care and resettlement support to displaced Palestinian families, including plans to sponsor 3000 orphans and treat 1500 individuals requiring medical attention.^12^ The psychological effects of chronic conflict on Palestinian children have been documented in multiple studies over the past two decades. In one of the earliest investigations, Thabet and Vostanis found that 21.5% of children in Gaza reported significant anxiety symptoms linked to social adversities and exposure to violence.^13^ Building on this work, Thabet, Abed and Vostanis examined the mental health outcomes of Palestinian refugee children aged 9 to 15 years and reported that exposure to war-related trauma was significantly associated with elevated symptoms of both PTSD and depression, with trauma exposure emerging as a stronger predictor of depressive symptoms.^14^ These studies identify the cumulative psychological burden faced by children living under occupation and repeated cycles of violence.

Despite the extensive documentation of mental health challenges among children in conflict zones, there remains a critical gap in understanding the psychological impact on Palestinian children recently displaced to host countries. This study seeks to address that gap by evaluating the prevalence and severity of anxiety and depression among war-displaced Palestinian refugee children and adolescents currently residing in Qatar.

## Method

### Study design and population

This study employed a cross-sectional design to assess symptoms of anxiety and depression among war-displaced Palestinian children in Qatar who had relocated from Gaza following the events of 7 October 2023. The study was conducted within the residential compound in Al-Thumama designated for the displaced Palestinian families. A total of 350 children aged between 8 and 18 years were recruited using convenience sampling. Participants were eligible if they were (a) of Palestinian descent, (b) currently residing in Qatar due to displacement from war zones and (c) able to provide assent along with parental/guardian consent. Exclusion criteria included cognitive impairment precluding valid questionnaire completion.

Informed written consent was obtained from the parents or legal guardians of all participants before enrolment. In addition to parental consent, informed written assent was sought from each child participant per ethical standards for research involving minors. The nature and purpose of the research were explained in detail, including the voluntary nature of participation and the right to withdraw at any point without consequence.

Participants were assured that their responses would remain confidential. No personally identifiable data were collected or linked to questionnaire responses. All data were anonymised using serial codes. These measures ensured that participants’ privacy and confidentiality were fully maintained throughout the study. Potential risks associated with participation, including psychological discomfort related to the trauma-related questions, were communicated to caregivers. Provisions were made to refer participants to mental health support services if distress arose during data collection.

As compensation, each participant received a 50 Qatari Riyal (approximately 13 USD) grocery voucher. This was intended purely as a humanitarian gesture to acknowledge the time and effort of Palestinian children and young people who had relocated to Qatar in extraordinary circumstances.

### Measures

Psychological symptoms were assessed using validated self-report instruments: The Screen for Child Anxiety Related Emotional Disorders-Child Version (SCARED-C) and the Short Mood and Feelings Questionnaire-Child Version (SMFQ-C).

SCARED-C is a 41-item questionnaire that screens for childhood anxiety disorders, including generalised anxiety disorder (GAD), separation anxiety, social phobia, school phobia and panic disorder.^15^ Children rate each item on a 3-point Likert scale (0 = not true or hardly ever true, 1 = somewhat true or sometimes true and 2 = very true or often true). Total scores ≥25 suggest clinically significant anxiety, with subscale cut-offs applied for specific anxiety domains. The tool demonstrates high internal consistency (Cronbach’s *α* = 0.91) and is validated in Arabic-speaking populations.^16,17^

The original MFQ is a 33-item child self-report version of the MFQ used to assess depressive symptoms over the past two weeks. Responses are rated as ‘true’, ’sometimes’, or ‘not true’, with total scores ranging from 0 to 66. Scores ≥27 are suggestive of clinically relevant depressive symptoms. The MFQ has strong psychometric properties and has been validated for use in both clinical and research settings among Arabic-speaking children and adolescents.^18^ The SMFQ was developed to enhance clinical and epidemiological use.^19^ It is a 13-item shortened version of the original 33-item version with parent and child versions.^20^ In the present study, we used the child self-report version of the SMFQ, which offers the advantages of brevity, simplicity and ease of administration.^21^ The self-reported and parent-reported versions of the MFQs both present a unifactorial structure and good psychometric properties.^22,23^

To collect background information and assess resilience-related factors, our team developed the Resilience and Demographic Questionnaire (RDQ) in Arabic. Initially compiled in English by experienced bilingual Child and Adolescent Psychiatrists, the questionnaire was translated into Arabic and then reverse-translated by independent bilingual colleagues to ensure linguistic accuracy and contextual integrity. It included items on age, gender, education level, living arrangements, family structure and exposure to traumatic events such as witnessing death or bombings. Resilience was conceptualised through questions on perceived family support, emotional coping strategies and duration of conflict exposure. The tool was piloted with five participants to assess clarity and cultural relevance, with no modifications deemed necessary.

All instruments were administered in Arabic by trained bilingual research team members. Questionnaires were anonymised using coded identifiers.

### Statistical analysis

Statistical analysis was conducted using SPSS for Windows, version 26.

For categorical variables, we calculated absolute and relative frequencies and the confidence intervals (for the prevalence of psychiatric disorders). For continuous variables, we calculated the mean, the s.d., as well as the confidence intervals of the mean.

To examine the factors associated with anxiety and depressive symptoms, we constructed multiple linear regression models with the SCARED-C and MFQ total scores respectively as dependent variables; and with the following variables as independent variables: age, gender, formal education, physical injury due to war, currently living with a parent, living with parents before the war, having witnessed the death of a first- or second-degree relative in Gaza, having witnessed the death of any person in Gaza, bombing in their living area and length of exposure to the war in Gaza ≥3 months.

For each of these multiple linear regression models, we determined the unstandardised regression coefficients (*β*), their confidence intervals, the partial coefficients (*r*) and the *p* values. Multiple linear regression assumptions (including linearity, normality of residuals, homoscedasticity and the absence of multicollinearity) were checked.

The defined significance level α was 0.05.

## Results

### Sociodemographic characteristics of participants (Table 1)

We included 350 participants with a mean age of 12.3 ± 2.9 years, with a gender ratio close to 1:1 (52% males, *n* = 182). The majority (98.3%, *n* = 344) had a formal education, and most (95.4%, *n* = 334) were living with their parents prior to the war. At the time of the study, around two-thirds (66.9%, *n* = 234) were still living with their parents. Around one third (33.7%, *n* = 118) sustained a physical injury during the war. Most (80.9%, *n* = 283) witnessed at least one death in Gaza, including the death of a first- or second-degree relative (67.4%, *n* = 236). Virtually all (99.4%, *n* = 348) respondents experienced bombing in their living area, and most were exposed to the war in Gaza for longer than one month (Table 1).

**Table 1:** Sociodemographic characteristics of participants

Variable	Results
Age, in years, mean (s.d.)	12.3 (2.9)
Gender, male, *n* (%)	182 (52.0)
Education level, *n* (%)	
Primary school	223 (63.7)
Secondary school	121 (34.6)
No formal education	6 (1.7)
Physical injury due to war, *n* (%)	118 (33.7)
Currently living with a parent, *n* (%)	234 (66.9)
Level of social support before war, *n* (%)	
Living with parents	334 (95.4)
Living with second-degree relatives	11 (3.1)
Other	5 (1.4)
Witnessed the death of a first- or second-degree relative in Gaza, *n* (%)	236 (67.4)
Witnessed the death of any person in Gaza, *n* (%)	283 (80.9)
Bombing in their living area, *n* (%)	348 (99.4)
Length of exposure to the war in Gaza, *n* (%)	
Less than one month	3 (0.9)
1–3 month(s)	89 (25.4)
4–6 months	133 (38.0)
More than 6 months	125 (35.7)

### Anxiety and depressive symptoms

**Fig. 1:**
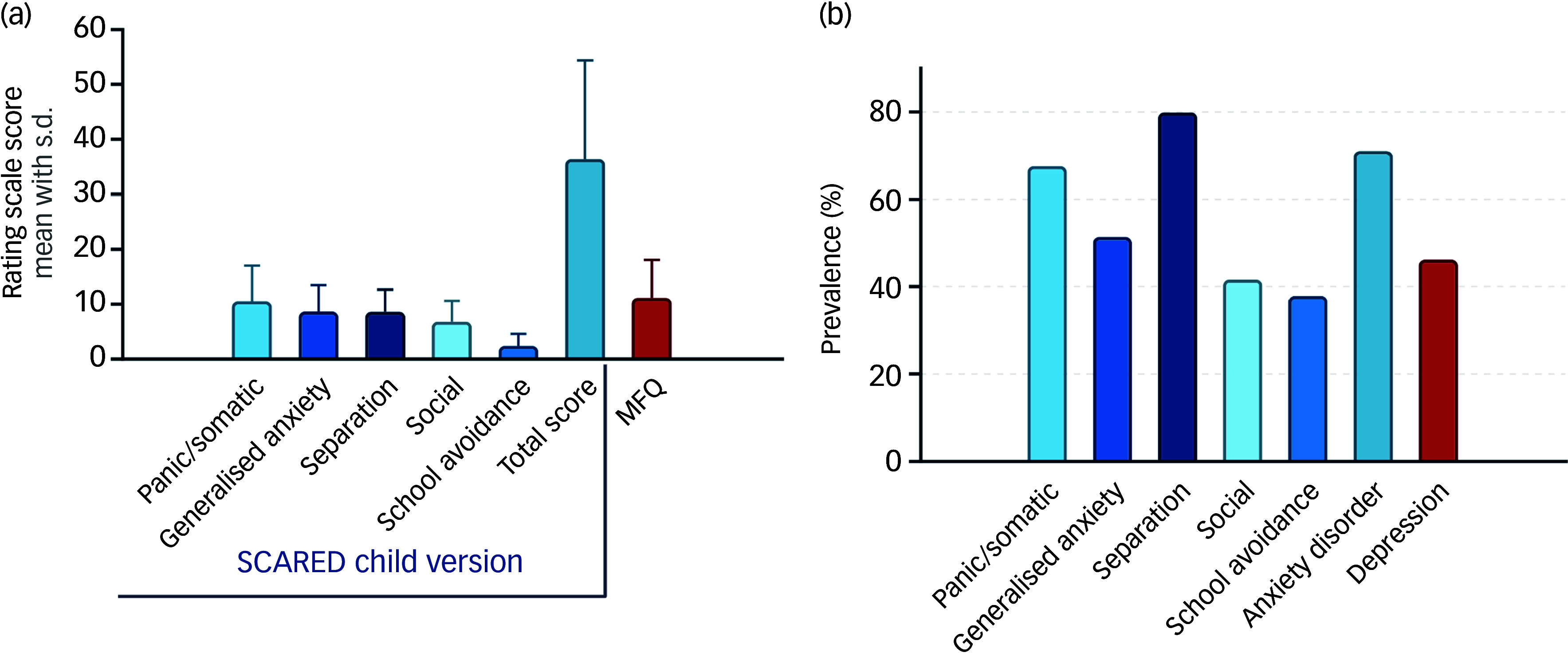
(a) Anxiety and depression scores. (b) Prevalence of anxiety disorders and depression. MFQ, Mood and Feelings Questionnaire; SCARED, Screen for Child Anxiety Related Emotional Disorders.

Figure 1(a) presents the mean rating scale scores and s.d. for various anxiety subtypes and depressive symptoms, as assessed by the SCARED-C and the MFQ, respectively. The mean total SCARED-C score, reflecting overall anxiety symptoms, was 36.3 ± 18.0 (95% CI: 34.4–38.2). The mean MFQ score, indicative of depressive symptoms, was 11.0 ± 6.9 (95% CI: 10.3–11.7).

As shown in Fig. 1(b), 70.9% (95% CI: 65.9–75.4%) of participants had a total SCARED-C score above the clinical threshold for an anxiety disorder, while 46.0% (95% CI: 40.8–51.2%) had MFQ scores suggestive of clinically significant depression. Among the specific anxiety subtypes, separation anxiety was the most prevalent, observed in 79.7% of the sample (95% CI: 75.3–83.7%).

### Factors associated with anxiety and depressive symptoms (Tables 2 and 3)

Anxiety symptoms were higher in females (*β* = 4.980 (1.258; 8.702); *p* = 0.009; *r* = 0.142) and those who witnessed the death of any person in Gaza (*β* = 7.400 (1.969; 12.831); *p* = 0.008; *r* = 0.144).

**Table 2:** Multiple linear regression analysis: factors associated with anxiety symptoms

Variable	*β*	*t*	*p*	95% CI for *β*		Partial correlation	VIF
				Lower bound	Upper bound		
(Constant)	31.534	2.046	0.041	1.225	61.844		
Age	−0.599	−1.819	0.070	−1.247	0.049	−0.098	1.029
Gender (female versus male)	**4.980**	**2.632**	**0.009**	**1.258**	**8.702**	**0.142**	**1.032**
Formal education	9.494	1.285	0.200	−5.043	24.031	0.070	1.062
Physical injury due to war	2.274	1.034	0.302	−2.053	6.602	0.056	1.248
Currently living with a parent	−1.378	−0.676	0.500	−5.389	2.633	−0.037	1.063
Social support before war – living with parents	−8.969	−1.918	0.056	−18.168	0.231	−0.104	1.101
Witnessed the death of a first- or second-degree relative in Gaza	2.434	1.058	0.291	−2.091	6.958	0.057	1.341
Witnessed the death of any person in Gaza	**7.400**	**2.680**	**0.008**	**1.969**	**12.831**	**0.144**	**1.362**
Bombing in the area	−0.155	−0.012	0.990	−25.137	24.828	−0.001	1.058
Length of exposure to the war in Gaza ≥3 months	−3.250	−1.397	0.163	−7.825	1.325	−0.076	1.210

VIF, variance inflation factor. Model: *R* ^2^ = 0.087; Adjusted *R* ^2^ = 0.060; *p* < 0.001.

Bold indicates statistically significant findings.

**Table 3:** Multiple linear regression analysis: factors associated with depressive symptoms

Variable	*β*	*t*	*p*	95% CI for *β*		Partial correlation	VIF
				Lower bound	Upper bound		
(Constant)	−0.667	−0.117	0.907	−11.847	10.513		
Age	0.235	1.930	0.054	−0.004	0.474	0.104	1.029
Gender (female versus male)	0.440	0.630	0.529	−0.933	1.813	0.034	1.032
Formal education	**7.489**	**2.747**	**0.006**	**2.127**	**12.851**	**0.148**	**1.062**
Physical injury due to war	**1.980**	**2.440**	**0.015**	**0.384**	**3.576**	**0.131**	**1.248**
Currently living with a parent	−0.019	−0.025	0.980	−1.498	1.460	−0.001	1.063
Social support before war – living with parents	**−4.655**	**−2.698**	**0.007**	**−8.049**	**−1.262**	**−0.145**	**1.101**
Witnessed the death of a first- or second- degree relative in Gaza	0.736	0.867	0.386	−0.933	2.405	0.047	1.341
Witnessed the death of any person in Gaza	**4.310**	**4.232**	**<0.001**	**2.307**	**6.313**	**0.224**	**1.362**
Bombing in the area	1.686	0.360	0.719	−7.529	10.901	0.020	1.058
Length of exposure to the war in Gaza ≥3 months	−1.494	−1.741	0.083	−3.181	0.194	−0.094	1.210

VIF, variance inflation factor. Model: *R* ^2^ = 0.161; Adjusted *R* ^2^ = 0.136; *p* < 0.001.

Bold indicates statistically significant findings.

Depressive symptoms were higher in those with a formal education (*β* = 7.489 (2.127; 12.851); *p* = 0.006; *r* = 0.148), those who had a physical injury during the war (*β* = 1.980 (0.384; 3.576); *p* = 0.015; *r* = 0.131), those who used to live *not* with their parents before war (*β* = −4.655 (−8.049; −1.262); *p* = 0.007; *r* = −0.145) and in those who witnessed the death of any person in Gaza (*β* = 4.310 (2.307; 6.313); *p* < 0.001; *r* = 0.224).

## Discussion

This cross-sectional study demonstrates the psychological impact of war-related trauma on the displaced Palestinian refugee youth. Of the participants, 70.9% had clinically significant anxiety symptoms, while 46% had signs suggestive of depressive disorders. These findings align with previous research conducted in Palestinian territories,^24–26^ where depression prevalence has been reported between 40 and 55.6%, and anxiety prevalence has ranged from 33% to as high as 94.9%, the latter reflecting particularly high variability. Such variability may be attributed to differences in trauma type and severity, methodological approaches, the circumstances of the participants at the time of the study and the varied use of assessment tools.

Our findings are also consistent with global data from conflict-affected regions, which similarly report elevated rates of psychiatric morbidity among war-exposed children. For instance, a systematic review and meta-analysis by Attanayake et al found pooled prevalence rates of 43% for depression and 27% for anxiety among 7920 war-exposed children, primarily from the Middle East.^27^ Kien and colleagues reported similarly high rates among young refugees and asylum seekers in Europe,^28^ while Henkelmann and colleagues found pooled prevalence estimates of 32% for anxiety and 28% for depression among refugee children and adolescents.^29^ More recently, in the context of the war in Ukraine, a study reported that 33.3% of adolescents in the conflict-affected Donetsk region exhibited anxiety symptoms, while 35.7% showed signs of depression.^30^ The study further demonstrated that adolescents in Donetsk were nearly three times more likely to experience severe anxiety and twice as likely to report moderate-to-severe depression compared to peers from non-conflict regions.

Among the various anxiety subtypes assessed, separation anxiety emerged as the most prevalent, affecting nearly 80% of the sample. This is consistent with prior literature showing heightened separation anxiety in children exposed to conflict-related displacement and disasters.^31,32^ Such symptoms likely reflect the significant attachment disruptions and instability associated with forced displacement, particularly when children are separated from familiar caregivers, communities and routines.

Girls reported significantly higher anxiety scores than boys, a trend echoed in other post-conflict contexts where girls reported higher levels of separation anxiety,^33^ depression^34^ and general psychological symptoms.^35^ This gender difference may be partly explained by sociocultural norms. Boys are often socialised to suppress emotional expression and adopt stoic behaviour, thereby potentially leading to underreporting of distress.^36^

Witnessing the death of any individual in Gaza emerged as the strongest predictor of both anxiety and depressive symptoms, exceeding the psychological impact of physical injury or length of exposure to the conflict. Witnessing violence during childhood has been associated with profound and enduring psychological sequelae, including regression, somatisation, self-blame and social withdrawal.^37^ Recent evidence reinforces this, highlighting that exposure to death during war constitutes a severe early-life trauma with long-term consequences, including increased risk of suicidal ideation.^38^ The psychological toll of such visual trauma is particularly detrimental in children and adolescents, whose emotional regulation and coping strategies are still developing.

A noteworthy and somewhat counterintuitive finding was the positive association between formal education and elevated depressive symptoms. Several interrelated factors may help explain this relationship. Participants with access to education are typically older and more cognitively mature, and therefore more capable of processing loss, disruption and the existential implications of war. Educational aspirations may also be derailed by displacement, further contributing to feelings of hopelessness. Moreover, the prevalence of depression has been shown to increase with age, particularly during and after puberty,^39^ offering a developmental explanation for this association.

In interpreting these findings, it is also important to consider the role of resilience. Although the RDQ used in this study was not a validated resilience scale, it provided important protective contextual insights into pre-war support and chronicity of adversity. Further investigation should aim to validate resilience tools for displaced Palestinian youth, as resilience factors are known to buffer against adverse mental health outcomes in conflict-affected populations.^40^

These findings have important implications for clinical practice, humanitarian response and policy development. There is a clear need to implement trauma-informed, culturally adapted mental health services for war-displaced refugee children, with particular attention to bereavement, separation anxiety and the heightened vulnerabilities observed among girls. The justification for culturally grounded approaches lies in evidence that interventions ignoring sociocultural context often fail to engage communities, whereas culturally integrated models improve both acceptability and outcomes.^41^ Integrating such services into schools, primary healthcare settings and community-based programmes may enhance accessibility and continuity of care. The host country context is also an important consideration. War-displaced children’s psychological outcomes are shaped not only by trauma exposure but also by the quality of education, healthcare and integration support available in the host setting. The post-war environment serves as a strong ecological context that can either support or hinder youth resilience by influencing access to protective resources such as community acceptance, family stability and educational opportunities.^40^

In Qatar, both governmental and non-governmental entities have initiated programmes aimed at supporting the educational and psychosocial needs of displaced children. However, sustained investment and targeted support are required to address the long-term mental health needs of displaced children, especially those separated from family or with relatives remaining in high-risk environments.^42^ Future research should prioritise longitudinal designs to clarify the trajectory and persistence of psychological symptoms over time, particularly in relation to time away from the conflict zone, as prior evidence in adults indicates that PTSD and depression can become chronic conditions, persisting for years or even decades, depending on ongoing stressors and length of displacement. Qualitative research may also enrich understanding by capturing children’s lived experiences, coping strategies and perceptions of care in culturally grounded terms. Finally, rigorous evaluations of intervention effectiveness and the development of context-specific, scalable mental health models are essential to inform evidence-based responses in similar humanitarian settings.

This study possesses several methodological strengths. It is among the first to examine the mental health of Palestinian children recently displaced to a host country, thereby contributing new empirical data to a critical yet understudied population. The use of validated and culturally adapted screening instruments (SCARED-C and SMFQ) ensures robust measurement of anxiety and depression symptoms. The large sample size enhances statistical power and allows for subgroup analyses across different age groups and trauma exposures. Furthermore, the inclusion of a comprehensive demographic and resilience questionnaire enabled a more nuanced examination of psychosocial variables relevant to mental health outcomes in war-displaced refugee children.

Nonetheless, a few limitations should be acknowledged. First, the cross-sectional design precludes any determination of causality and limits understanding of symptom progression over time. Longitudinal studies are required to assess changes in mental health status, treatment responsiveness and long-term adaptation. Second, reliance on self-report measures may be subject to bias, including underreporting of distress, particularly among boys, due to social desirability or internalised cultural norms regarding emotional expression. Third, the convenience sampling from a single residential compound may limit generalisability, as the experiences and access to services in this setting may not reflect those of displaced children residing in different host countries. Additionally, severely distressed children may have been less likely to participate, possibly leading to an underestimation of prevalence rates. Finally, while the RDQ provided valuable socioecological context, it has not been formally validated and requires further psychometric evaluation. Moreover, as a structured questionnaire rather than a qualitative tool, it cannot adequately capture the culturally specific concept of *sumud*, a form of steadfastness rooted in ideology, connection to the land and persistence in struggle, which is central to understanding resilience and well-being among Palestinian youth.^43^

### Future directions

This study reveals alarmingly high rates of anxiety and depressive symptoms among war-displaced Palestinian refugee children and adolescents resettled in Qatar, highlighting the profound psychological toll of armed conflict, forced displacement and exposure to death and destruction. Separation anxiety emerged as the most prevalent anxiety subtype, and witnessing death was the most significant predictor of both anxiety and depressive symptoms. These findings echo a consistent body of international evidence highlighting the elevated mental health burden borne by children and adolescents affected by war. While our research contributes valuable insights into the mental health needs of war-displaced Palestinian youth, its implications go far beyond clinical concern. The enduring and cumulative trauma experienced by these children cannot be addressed through mental health interventions alone. It demands urgent global action, most critically, an immediate end to the violence in the region. No amount of psychological support can substitute for safety and the right of children to live free from fear. Mental health professionals must advocate not only for trauma-informed care and psychosocial support but also for justice, protection and peace.

## Data Availability

The data that support the findings of this study are not publicly available due to ethical and privacy restrictions.
