# Mental health burden of conflict: rates and correlates of depressive and anxiety symptoms among displaced Palestinian children and adolescents in Qatar – ERRATUM

**DOI:** 10.1192/bjo.2025.10961

**Published:** 2026-01-07

**Authors:** Mohamed Adil Shah Khoodoruth, Yahia Albobali, Olfa Selmi, Sami Ouanes, Marwan Abdelkarim Ali Abdelkarim, Areeg Hassan Mohamed Elhassan, Menatalla Abdelkader, Taieb Turki, Ahmed Abdelhakim Ahmed Elzok, Abdul Waheed Khan, Majid Alabdulla, Yasser Saeed Khan

**Keywords:** Anxiety disorders, depressive disorders, war, displacement, children, erratum

## Abstract

**Figure:**
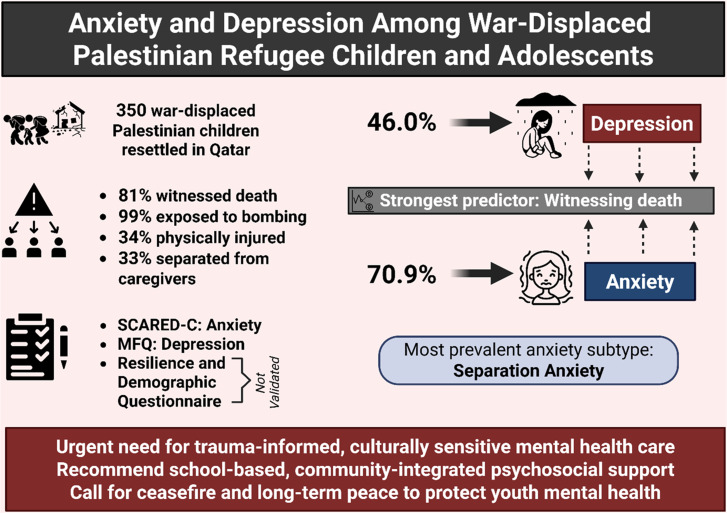

In the original publication of this article, the graphical abstract had typographical errors.

This has since been updated on the article. The Publisher apologises for this error.
